# Role of GntR Family Regulatory Gene* SCO1678* in Gluconate Metabolism in* Streptomyces coelicolor* M145

**DOI:** 10.1155/2017/9529501

**Published:** 2017-04-27

**Authors:** Olga Tsypik, Roman Makitrynskyy, Agnieszka Bera, Lijiang Song, Wolfgang Wohlleben, Victor Fedorenko, Bohdan Ostash

**Affiliations:** ^1^Department of Genetics and Biotechnology, Ivan Franko National University of Lviv, Lviv 79005, Ukraine; ^2^Microbiology and Biotechnology, Interfaculty Institute of Microbiology and Infection Medicine, University of Tübingen, Tübingen, Germany; ^3^Department of Chemistry, University of Warwick, Coventry CV4 7AL, UK

## Abstract

Here we report functional characterization of the* Streptomyces coelicolor* M145 gene* SCO1678,* which encodes a GntR-like regulator of the FadR subfamily. Bioinformatic analysis suggested that* SCO1678* is part of putative operon* (gnt)* involved in gluconate metabolism. Combining the results of* SCO1678* knockout, transcriptional analysis of* gnt* operon, and Sco1678 protein-DNA electromobility shift assays, we established that Sco1678 protein controls the gluconate operon. It does so via repression of its transcription from a single promoter located between genes* SCO1678* and* SCO1679*. The knockout also influenced, in a medium-dependent manner, the production of secondary metabolites by* S. coelicolor*. In comparison to the wild type, on gluconate-containing minimal medium, the* SCO1678* mutant produced much less actinorhodin and accumulated a yellow-colored pigment, likely to be the cryptic polyketide coelimycin. Possible links between gluconate metabolism and antibiotic production are discussed.

## 1. Introduction

Bacteria of genus* Streptomyces* are abundant soil dwellers having unparalleled capacity to produce bioactive small molecules and to assimilate complex plant (e.g., lignins and cellulose) and animal polymers [1]. These properties fuel interest in* Streptomyces* as a source of novel drug candidates [2], valuable hydrolytic enzymes [3], and ecofriendly plant protection strategies [4]. Streptomycetes have evolved a complicated regulatory network that coordinates their primary metabolism with biosynthetic pathways responsible for the production of specialized secondary metabolites and breakdown of polymers [5, 6]. Core regulators of primary metabolism of streptomycetes are intimately linked to antibiotic production [7].* Streptomyces* genomes are also very large, between 6 and 11 Mbp, and harbor a lot of regulatory genes. The overall understanding of how these regulatory genes control transcription is still unclear [8]. This limits our current ability to take full advantage of genomic potential of* Streptomyces* for its biomedical and industrial applications. Therefore, it is important to continue functional characterization of various regulatory genes in well-known model species, like the best studied* Streptomyces coelicolor* A3(2) (or its derivative M145).

Recently we have carried out extensive in silico analysis of GntR family transcriptional factors, one of the biggest and yet poorly understood groups of regulators in* Streptomyces* [9]. As a result, twelve GntR regulators have been described that are conserved across the Streptomycetaceae family. We refer to them as “core GntRs” to highlight their potential important function in* Streptomyces* biology. Two of them, WhiH and DasR, have been extensively studied in the past. WhiH controls early steps of sporulation [10, 11], while DasR is a pleiotropic regulator of multiple carbohydrate transporters, chitin metabolism and antibiotic production genes [12]. A third regulator, Sco0823, was recently proposed by us to participate in ferric ion uptake [9]. Functions of the nine other core GntRs are yet to be explored. Here we report functional characterization of* S. coelicolor* M145 gene* SCO1678*, which encodes a GntR type regulator from the FadR subfamily. We demonstrate that* SCO1678* encodes a repressor of the gluconate operon and that the promoter of gluconate kinase gene* SCO1679* is the main target of Sco1678 regulatory action. Interestingly, a knockout of* SCO1678* also altered the secondary metabolite profile of* S. coelicolor*. Our work reveals one more regulatory checkpoint that links primary metabolism and antibiotic production in actinomycetes.

## 2. Materials and Methods

### 2.1. Bacterial Strains and Culture Conditions

Bacterial strains used in this work are listed in Table 1.* E. coli *DH5*α*, ET12567 (pUZ8002), and BW25113 (carrying pIJ790) were used for routine cloning, to perform intergeneric conjugation with* Streptomyces* species and to carry out RedET-mediated gene replacement, respectively [13].* E. coli* DH5*α* and ET12567 (pUZ8002) were grown at 37°C in Luria-Bertani (LB) medium [14]; strain BW25113 was grown at 28°C in 2x YT medium (Tryptone: 20 g, Yeast extract: 10 g, and sodium chloride: 5 g per 1 l of distilled water). All* Streptomyces* strains were grown at 28°C. Solid SFM medium (soya flour, mannitol, agar—20 g/liter each) was used to harvest* Streptomyces* spores and to plate* Streptomyces*-*E. coli* matings. To analyze the gene transcription profile, precultures of* Streptomyces* strains were grown in TSB medium for 24 h, and then mycelium was harvested by centrifugation, washed three times with water, and inoculated into SMM medium with 1% glucose or gluconate for 36 h [15]. To analyze antibiotic (actinorhodin (ACT), undecylprodigiosin (RED), and coelimycin (CPK)) production, liquid YMPG, R2YE, SMM, and Oxoid agar media, respectively, were used [15, 16]. Where needed, media were supplemented with respective antibiotics.

### 2.2. DNA Techniques

Isolation of plasmid DNA from* E. coli* and chromosomal DNA from* Streptomyces*, DNA digestion by restriction endonucleases, agarose gel electrophoresis, and DNA ligation were performed using standard protocols [14, 15].* E. coli* transformation and intergenetic* E. coli-Streptomyces* matings were performed as described in [15]. DNA amplification by PCR was generated with Taq (NEB) and Phusion (NEB) DNA polymerases. All plasmids were verified by DNA sequencing.

### 2.3. Construction of pGUS-gntRp Plasmid

Approximately 230-bp of the promoter region of* gntR* was amplified with primers SCO1678gusXbaI and SCO1678gusKpnI, digested, and cloned into XbaI and KpnI sites of pGUS-vector giving pGUS-gntRp. Oligonucleotides used throughout this work are listed in Table 2.

### 2.4. Construction of pKC-gntR Overexpression Plasmid and pSET-gntR for Complementation

*SCO1678 (*=*gntR)* coding sequence with 230-bp promoter region was amplified with SCO1678 cmpl-f/SCO1678 cmpl-r primer pair. PCR product was digested with BamHI and XbaI and cloned into respective sites of moderate copy number vector pKC1139 and integrative plasmid pSET152, giving pKC-gntR and pSET-gntR, respectively.

### 2.5. Construction and Verification of* SCO1678* Knockout Strain

An in-frame deletion mutant* S. coelicolor* ΔgntR was constructed using REDIRECT technology [13]. For this purpose, gene* SCO1678* with 3-kb flanking regions was amplified from chromosomal DNA of* S. coelicolor* with the following primers: SCO1678-f and SCO1678-r. The PCR product was digested with HindIII and XbaI restriction endonucleases and subsequently cloned into respective sites of pKC0702. The obtained plasmid pKC0702-SCO1678 was transformed into* E. coli* BW25113 where replacement of* SCO1678* by apramycin resistance cassette* aac(3)IV* was accomplished. The latter was amplified from plasmid pLeere using SCO1678_acc_f and SCO1678_acc_r primer pair. The knockout plasmid pKC0702-SCO1678::Am was introduced into the wild type* S. coelicolor* M145 followed by screening of apramycin-resistant and hygromycin-sensitive colonies. Positive clones were indicative that a double crossover had occurred between the homologous regions of the M145 genome and on the knockout construct. Markerless mutant* S. coelicolor *ΔgntR was generated by implication of site-specific recombinase Cre as described in [18]. The* SCO1678* disruption and marker eviction were confirmed via PCR (primers SCO1678 cmpl-f/SCO1678 cmpl-r).

### 2.6. RT-PCR

RNA for semiquantitative RT-PCR was isolated using RNeasy mini kit (Qiagen) according to recommendations of the supplier. RNA samples were checked for DNA contamination by PCR. For cDNA synthesis 3 *μ*g of total RNA was incubated with random primers for five minutes at 72°C. The remaining components (RNase inhibitor, dNTPs, reverse transcriptase buffer, DTT, and ProtoScript II reverse transcriptase (NEB)) were subsequently added and reverse transcription (RT) was carried out at 42°C for 60 min. 200 ng of synthesized RT products was used as a template for subsequent PCR analysis with primers listed in Table 2. Obtained PCR products were separated on a 1.5% agarose gel to analyze the transcription profile of genes of interest.

### 2.7. Production and Purification of His-Tagged SCO1678 Protein

To produce C-terminally hexahistidine-tagged Sco1678 protein (GntR-His), its ORF was cloned into pET28a expression vector using PCR and primers SCO1678NcoI-f and SCO1678HindIII-r. Resulting plasmid was labeled as pET28a-SCO1678. For GntR-His production,* E. coli* BL21 (DE3) GOLD carrying pET28a-SCO1678 was grown in LB supplemented with tetracycline and kanamycin until OD_600_ reached 0.5; then the culture was induced with 1 mM IPTG (isopropylthiogalactoside) and incubated for six hours at 22°C. Cells were collected by centrifugation and resuspended in a lysis buffer (50 mM Na_2_HPO_4_, 300 mM NaCl, and 20 mM imidazole, pH 7) containing proteinase inhibitor (Roche). Cells lysis was achieved by two consecutive passages through a French press (American Instrument Corporation) at 1000 psi. The cell lysate was centrifuged at 18000 rpm for 30 minutes and soluble fraction was applied to Ni-NTA agarose resin (Qiagen), washed two times with wash buffer (50 mM Na_2_HPO_4_, 300 mM NaCl, and 40 mM imidazole, pH 7). The protein was eluted with 200 mM imidazole and dialyzed against storage buffer (50 mM Na_2_HPO_4_, 300 mM NaCl, and 5% glycerol, pH 7). Protein concentration was determined by Bradford assay.

### 2.8. Electromobility Shift Assay (EMSA) of DNA-Protein Complexes

Putative promoter regions of* SCO1263*,* gntP*,* gntZ,* and* gntR-K* were amplified from chromosomal DNA of* S. coelicolor* with primers listed in Table 2 and subsequently labeled with indocarbocyanine (Cy5) as described in [19]. Cy5-labeled probes (0.2 pmol) were incubated with purified recombinant GntR-His (see above) in concentrations of 0.93 to 4.18 pmol in binding buffer (20 mM Tris/HCl pH 7.5, 50 mM KCl, 10 mM MgCl_2_, 5% (v/v) glycerol, and 0.5 mM EDTA) for 25 min at 25°C. Electrophoresis was carried out in 8% native polyacrylamide gel in 1x TBE buffer at 150 V for 60 min. DNA bands were visualized by fluorescence imaging using a Typhoon Trio variable mode imager (GE Healthcare). EMSA was used to test whether gluconate, glucono-1,5-lactone, and glucose are potential effector molecules for Sco1678 (at final concentration of 5 mM in reaction mixture).

### 2.9. Analysis of *β*-Glucuronidase Activity

Strains carrying pGUS-gntR or pGUS plasmids were grown for 36 h in SMM supplemented with certain carbon source. Transcription level of *β*-glucuronidase from* gntR* promoter was examined according to [17].

### 2.10. Analysis of Antibiotic Production

ACT and RED production levels were quantified as described in [15], in YMPG and R2YE media, respectively. ACT production was analyzed in SMM medium with either glucose or gluconate as the sole carbon source.

## 3. Results

### 3.1. Analysis of gnt Operon in* S. coelicolor*

**Figure 1:**
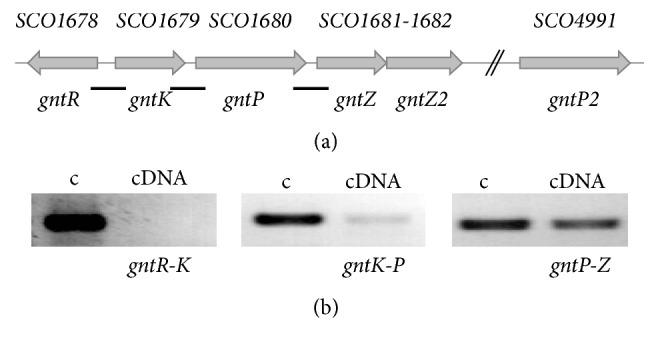
Genes for gluconate operon in* S. coelicolor* (a) and their transcriptional organization (b). Three black rectangles beneath the* SCO* genes indicate fragments amplified during RT-PCR analysis of intergenic regions (see (b)).

Gene* SCO1678* encodes a 233 aa transcriptional factor from the GntR family of regulators. As a member of this family Sco1678 protein consists of a N-terminal DNA-binding domain with GntR-like helix-turn-helix motif followed by a C-terminal effector binding/oligomerization domain. Based on the secondary structure of the C-terminal domain, Sco1678 was proposed to fall into the FadR subfamily of GntRs [9]. Divergently to* SCO1678*, genes of putative gluconate* (gnt)* operon are located (Figure 1(a)). Gene* SCO1679 (gntK)* encodes gluconokinase that phosphorylates gluconate to glucono-5-phosphate which is then metabolized in the pentose phosphate pathway. Gene* SCO1680 (gntP)* encodes gluconate permease that transports the molecule into a cell. The next two genes,* SCO1681*-*SCO1682 (gntZ*-*gntZ2)*, overlap by 4 nucleotides and encode gluconate dehydrogenase and zinc-binding alcohol dehydrogenase, respectively. RT-PCR confirmed transcriptional coupling of genes* SCO1679-1680-1681* (Figure 1(b)).

Another carbohydrate transport membrane protein Sco4991 shows 39% identity to GntP permease of* Bacillus subtilis* and 38% to* S. coelicolor* gluconate permease Sco1680. The former, therefore, might be involved in gluconate uptake as well. This agrees with recent global analysis of the entire array of transport proteins in* S. coelicolor*, where Sco1680 and Sco4991 were annotated as high-affinity gluconate permease and gluconate permease, respectively [20].

### 3.2. Expression and Knockout of* SCO1678*

**Figure 2:**
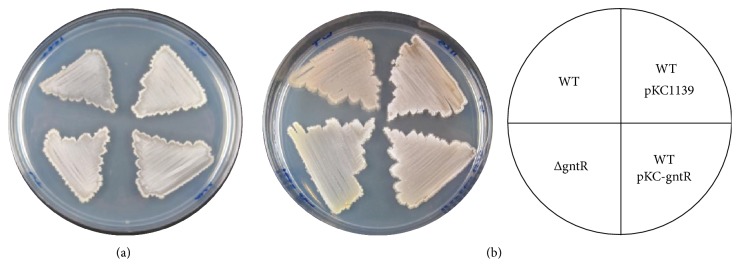
*S. coelicolor* strains grown on MM with glucose (a) or gluconate (b). WT:* S. coelicolor *M145; ΔgntR:* S. coelicolor* ΔgntR; WT pKC-gntR:* S. coelicolor* harboring pKC-gntR; WT pKC1139:* S. coelicolor* plus empty vector pKC1139.

Organization and regulation of* gnt* operons in* Escherichia coli* and* Bacillus subtilis* are well studied [21, 22]. In both cases transcription of* gnt *genes is repressed by GntR, a protein that served as a prototype for the entire GntR family of regulators. From available in silico data we propose that* SCO1678* also encodes GntR. If, contrary to our assumption, SCO1678 encodes an activator of* gnt* genes, then its deletion would lead to arrested or significantly reduced growth in presence of gluconate as the sole carbon source. To probe function of Sco1678, the* SCO1678* gene was overexpressed on a moderate copy number plasmid pKC1139 and in-frame deletion mutant* S. coelicolor *ΔgntR was generated. Both strains as well as the wild type were grown in liquid SMM or on MM-agar plates supplemented with either glucose or gluconate (1%, w/v). No differences in growth rate and sporulation were detected among the strains (Figure 2). Complementation of ΔgntR with* SCO1678* (plasmid pSET-gntR) also had no recognizable effects on growth. Our results agree with the assumption that* SCO1678* encodes a repressor of the* gnt *genes. We noted, though, that ΔgntR on gluconate-containing medium had different coloration, probably because of changes in secondary metabolism, as discussed below (see Section 3.7).

### 3.3. Transcriptional Profile of gnt Genes

**Figure 3:**
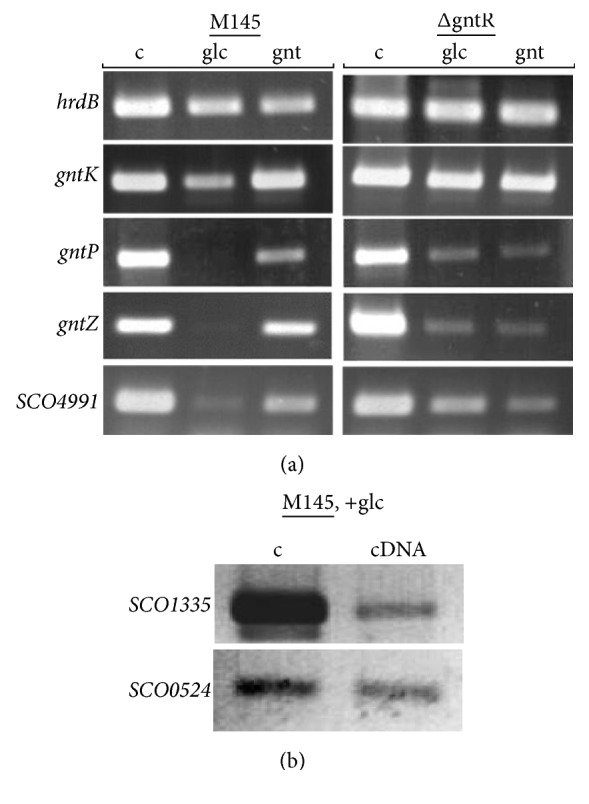
Transcriptional profile of (a)* gnt *genes in* S. coelicolor* M145 (M145) and ΔgntR (ΔgntR), and (b)* SCO1335* and* SCO0524* genes possibly related to pentose phosphate pathway. As a template for RT-PCR chromosomal DNA (c) and cDNA obtained from strains grown in either glucose (glc) or gluconate- (gnt-) containing SMM were used.

To further elucidate* SCO1678* function, expression of the* gnt* operon of* S. coelicolor* was investigated by RT-PCR. For this purpose, a wild type strain and* S. coelicolor *ΔgntR were grown in liquid SMM medium supplemented with either glucose or gluconate as a sole carbon source. Data are summarized in Figure 3. We note here that our analysis was not quantitative, and transcription level can only be roughly compared within one strain. Transcription of* gntK*,* gntP*,* gntZ,* and* SCO4991* in wild type strain M145 when cultured in gluconate-containing medium indicates their involvement in gluconate metabolism and transport. However, transcription of* gntK* and* SCO4991* was also detected in M145 in presence of glucose, although their level was lower comparing to that in presence of gluconate. Further analysis of expression of* gnt* gene in* S. coelicolor *ΔgntR revealed that in presence of either tested sugar, all four gene transcriptions were detectable.

Absence of* gntP*,* gntZ* transcription, and reduced level of* gntK* transcription in presence of glucose and transcription of all four genes in the presence of gluconate implied that Sco1678 is a gluconate-dependent repressor for the transcription of* gnt* genes. Transcription of* gntK* and* SCO4991* in the presence of glucose was puzzling. However, glucose can be converted into gluconate and gluconate-6-phosphate and further metabolized through the pentose phosphate pathway. In this case two enzymes are involved: glucose-1-dehydrogenase (Sco1335) converts glucose to glucono-1,5-lactone which is further metabolized to gluconate by gluconolactonase (Sco0524). These same molecules may induce* gnt* operon to some extent. Both aforementioned genes were expressed during wild type growth in glucose-containing SMM (Figure 3(b)). Transcription of* SCO4991* in presence of either carbohydrate implies that this transporter can be involved in uptake of not only gluconate but other sugars as well.

### 3.4. Binding of Recombinant Sco1678 Protein to Promoter Regions of Putative Target Genes

**Figure 4:**
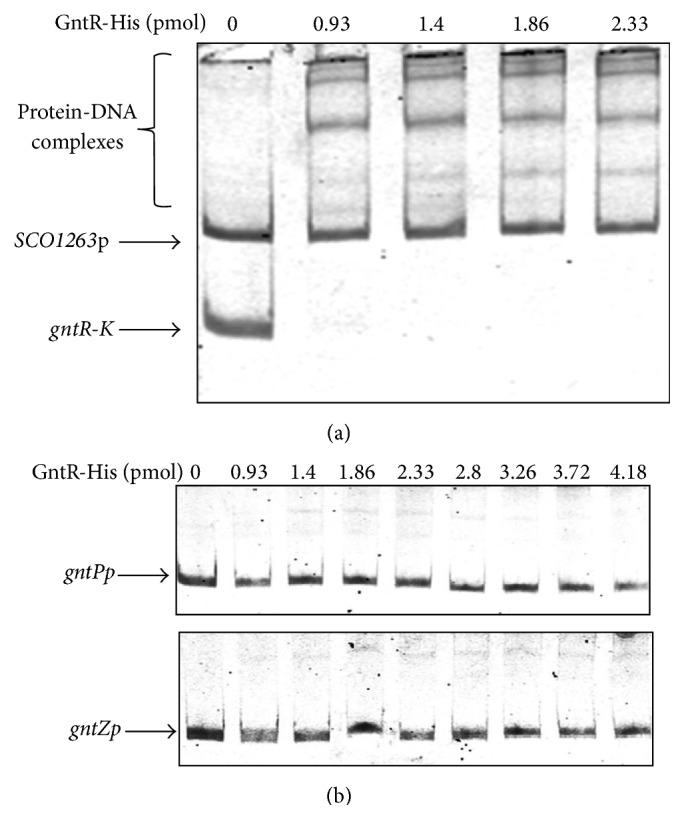
Binding of Sco1678 (GntR) to* gntR-K* region (a) and to promoters of* gntP* and* gntZ* (b). 0.2 pmol DNA fragment was incubated with indicated GntR-His concentration. Promoter region of* SCO1263* was used in reaction as a negative control to check GntR-His specificity to its target* (gnt)* genes.

Pure hexahistidine-tagged Sco1678 (GntR-His; 0.93 to 4.14 pmol) was incubated with Cy5-labeled DNA fragments encompassing promoters of* gntR-gntK, gntP*, and* gntZ, *and reaction products were separated in native acrylamide gel. As shown in Figure 4, GntR-His shifts* gntR-gntK* intergeneric region starting from a concentration of 0.93 pmol. No band shifts were observed with promoters of* gntP* and* gntZ* even when higher concentrations of the protein were applied. This agrees with our RT-PCR analysis of* gnt* operon (see Figure 1(b)), showing that* gntK, gntP*, and* gntZ* are transcribed as a polycistronic mRNA. Therefore, Sco1678 binds only to* gntR*-*gntK* region.

### 3.5. Identification of Sco1678 Effector Molecules

**Figure 5:**
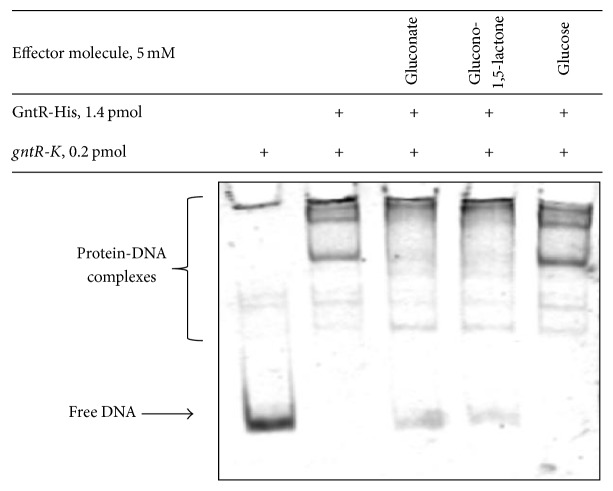
EMSA-mediated identification of putative effector molecules for recombinant Sco1678 protein. Binding of Sco1678 (1.4 pM) to* gntR-K* intergenic region was tested in presence of effectors mentioned in the figure. See Materials and Methods for workup conditions.

GntR type transcriptional factors act as repressors of gene transcription. Upon binding the appropriate effector molecule, the GntR repressors are no longer able to recognize promoters [23]. In most cases, such an effector molecule is a metabolite from the pathway where products of target genes are involved. To identify the putative effector molecule of GntR the DNA-binding shift assay was performed in the presence of gluconate and gluconolactone as well as glucose (the latter was used as a negative control). For this purpose, purified GntR-His was incubated with* gntR-K* intergeneric region in binding buffer that contains putative ligand (5 mM) and subsequently separated in a native acrylamide gel. As shown in Figure 5 gluconate and gluconolactone interfered with binding of GntR-His to the* gntR-K* region but could not release the DNA completely. The presence of glucose did not affect protein-DNA interaction.

### 3.6. Autoregulatory Function of* SCO1678*

**Figure 6:**
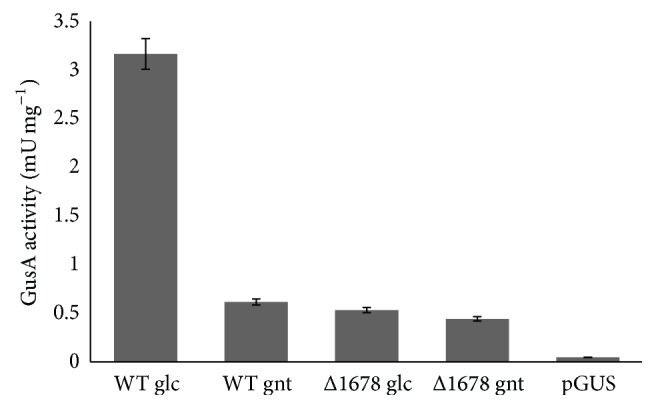
Transcriptional activity of* SCO1678 (gntR)* promoter in* S. coelicolor* WT (M145) and ΔgntR. The *β*-glucuronidase activity was measured from both strains carrying pGUS-gntR grown for 36 hours in SMM medium supplemented with glucose (WT glc, Δ1678 glc) or gluconate (WT gnt, Δ1678 gnt).* S. coelicolor* pGUS was grown in SMM with glucose and used as a control strain. Data represent mean values of three independent replicates. Error bars, ±2SD.

Most GntR regulators either repress or activate the transcription of their own genes. To explore this further, promoter of* SCO1678* was fused with* gusA* reporter gene encoding *β*-glucuronidase, and the resulting construct was introduced into both* S. coelicolor *ΔgntR and wild type strains. Data are summarized in Figure 6. Fivefold increased* SCO1678* transcription was observed in the wild type grown in SMM-glucose medium as compared to those grown in SMM-gluconate medium. No differences in transcription were observed for* SCO1678* deletion mutant grown in presence of either glucose or gluconate, and it was equal to the transcription level observed for the wild type grown on gluconate. Our data showed that Sco1678 upregulates its own gene transcription and that the* SCO1678* promoter is not activated in the presence of the effector molecule gluconate.

### 3.7. *S. coelicolor* ΔgntR Produces Yellow Polyketide Coelimycin

**Figure 7:**
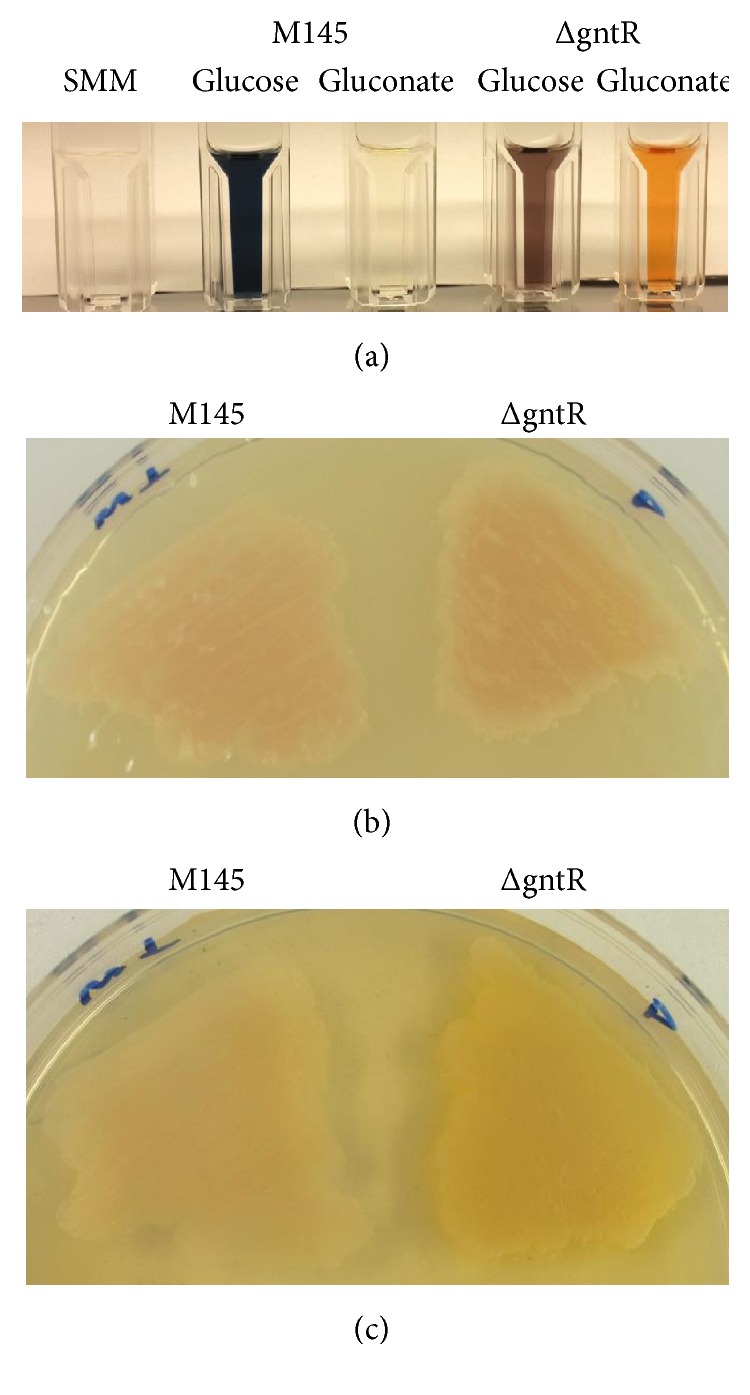
Gluconate inhibits ACT production in* S. coelicolor* M145 and triggers production of yellow-pigmented compound, CPK, in* S. coelicolor *ΔgntR. Spent media in cuvettes are shown (a) where respective strains were grown. The production levels correspond to equal amounts of biomass, as judged by Bradford protein assay. On glucose-containing SMM agar both M145 and ΔgntR produce ACT, and yellow pigment was not obvious (b), while on gluconate-containing Oxoid agar (c) these strains differ in secondary metabolism profile.

*S. coelicolor* produces prolifically at least five natural compounds: ACT, RED, methylenomycin, calcium-dependent antibiotic, and coelimycin [24]. Secondary metabolite production by* Streptomyces* is tightly linked to physiological and nutritional status. To investigate putative influence of* SCO1678* deletion on ACT and RED production, strains were grown in YMPG, R2YE, and SMM media supplemented with either glucose or gluconate. No differences in antibiotics production were observed in YMPG and R2YE media in comparison to the wild type. In glucose-containing SMM medium both strains produced ACT (Figure 7(a)). However, the level of its production was decreased in the presence of gluconate. Moreover, gluconate triggered the production of a yellow compound, most likely coelimycin (CPK), in* S. coelicolor *ΔgntR. Yellow pigment accumulation was also observed when strains were grown on gluconate-containing Oxoid agar plates (Figures 7(b) and 7(c)). Secondary metabolism profile (coloration of agar plates) reverted to wild type when* SCO1678* was introduced (on integrative plasmid pSET-gntR) into ΔgntR (data not shown). The identity of yellow pigment to CPK was established via analytical HPLC-MS analysis. The new peak (absent in extracts of M145) showed identical retention time (16.5 min) with standard Coelimycin P1, and based on the high resolution and high accurate MS data (349.1214 Da; (M+H)^+^), identical molecular formulae are generated as coelimycin P1; UV absorbance spectra of our peak and CPK were also the same.

## 4. Discussion

Streptomycetes can metabolize a variety of carbohydrates including gluconic acid. In this work, we address for the first time the genetic basis of the ability of model strain,* S. coelicolor*, to utilize gluconate. First, it is imported into the cell by gluconate permease (GntP = Sco1680, maybe Sco4991), after phosphorylation to gluconate-6-phosphate by gluconokinase (GntK = Sco1679) and it then enters the pentose phosphate pathway. Typically, genes responsible for gluconate uptake are organized into an operon under negative control of the GntR repressor [25] (see Figure 1). Transcription level of key* gnt* genes of* S. coelicolor* were high when grown in gluconate-containing medium and were absent or severely repressed when grown in the presence of glucose. These observations point to involvement of respective proteins in gluconate metabolism. By combining the results of* SCO1678* knockout, RT-PCR of* gnt* operon, and EMSA of Sco1678 protein, we can safely conclude that Sco1678 is repressor of* gnt* operon.

Sco1678 binds to promoter region of gluconokinase, but not permease or dehydrogenase, suggesting that these genes are transcribed as polycistronic mRNA. This hypothesis was confirmed by RT-PCR because we could amplify* gntK-P* and* gntP-Z* intergenic regions from cDNA (Figure 1(b)).

In SMM medium gluconate inhibits ACT production in both strains. This is not the first case where gluconate has abolished antibiotic production. For instance, cocultivation of* S. coelicolor* and* Pseudomonas fluorescens* BBc6R8, a producer of gluconic acid, stops ACT production [26]. Gluconate inhibits prodigiosin biosynthesis in* Serratia *sp. ATCC39006. In this case PigT regulator activates transcription of the biosynthetic operon* pigA–O*, but addition of gluconate decreases transcription of the latter. PigT shows high level homology to* E. coli* GntR protein [27]. Our working hypothesis is that gluconate, in the absence of the other carbon sources and regulatory function of Sco1678, serves as a metabolic signal that switches secondary metabolism from production of typical metabolites (ACT and RED) to minor or cryptic ones, such as coelimycin. We speculate that this secondary metabolic switch is mediated by a regulatory protein not yet known. Work is currently underway in our laboratories to experimentally explore this hypothesis.

## 5. Conclusions

Protein Sco1678, encoded within* Streptomyces coelicolor* M145 genome, belongs to twelve of the most conserved regulators of GntR family across class of Actinobacteria. Here we show for the first time that* SCO1678* gene encodes GntR, or repressor of gluconate utilization operon. Its repressor function is exerted via binding to single promoter upstream of gluconokinase gene* SCO1679* and is responsive to gluconate. We revealed that GntR in* S. coelicolor* constitutes a regulatory checkpoint for secondary metabolism, because under certain growth conditions the* SCO1678* knockout decreases actinorhodin titers and induces the production of otherwise cryptic polyketide coelimycin. Our data suggest that further studies of* SCO1678 (gntR)* are worth pursuing. They will lead to further insight into coordination of primary and secondary metabolic pathways and help devise novel approaches towards the induction of silent gene clusters in actinomycetes.

## Figures and Tables

**Table 1:** Bacterial strains and plasmids used in this work.

Strains or plasmids	Description	Source or reference
*S. coelicolor* M145	SCP1^−^, SCP2^−^ derivative of A3(2); ACT and RED producer	[15]
*S. coelicolor* ΔgntR	*SCO1678* knockout in M145	This work
*S. coelicolor* pKC-SCO1678	SCO1678 overexpression in M145	This work
*S. coelicolor* pKC1139	M145 with pKC1139 empty vector	This work
*S. coelicolor* pGUS	M145 carrying pGUS	This work
*S. coelicolor* pGUS-gntRp	M145 carrying pGUS-gntRp	This work
*Escherichia coli *DH5*α*	Routine cloning host	Life Technologies
*E. coli *ET12567 (pUZ8002)	Host for conjugative DNA transfer	[13]
*E. coli *BW25113 (pIJ790)	Host for recombineering experiments	[13]
*E. coli *BL21 (DE3) GOLD	Strain for recombinant protein production	Stratagene
pIJ790	ts-plasmid carrying genes for *λ*-RED recombination, CmlR	[13]
pLeere	Carrying *acc(3)IV* flanked by loxP-sites, apramycin and ampicillin resistances	Luzhetskyy
pKC1139	pSG5 *ts*-replicon; apramycin-resistant (AmR) shuttle vector	[15]
pKC-SCO1678	pKC1139 harboring SCO1678 with promoter region; *SCO1678* overexpression, AmR	This work
pKC0702-SCO1678	pKC0702 harboring SCO1678 with 3-kb flanking region, HygR	This work
pKC0702-SCO1678::Am	SCO1678 knockout construct; Δ*sco1678*::*hyg* (HygR and AmR)	This work
pGUS	Promoterless *gusA*-containing plasmid, apramycin resistance	[17]
pGUS-gntRp	pGUS with *gntRp*-*gusA* fusion, AmR	This work
pET28a	Protein expression vector, pET-system	Novagen
pET28a-SCO1678	Sco1678-6His protein expression	This work

**Table 2:** Primers used in this work.

Primer name	Sequence	Purpose/PCR product
SCO1678-f	AATAAAAGCTTCGTGACTGAAGAAGAGCGAA	To amplify *SCO1678* with 3-kb flanks
SCO1678-r	AATAATCTAGAAGGGAGTAATGAGGCTACGA	
SCO1678_acc_f	GACTTTTTGTTCCCGTGACATGCCCCGTACGCTGAGTGCATGGATATCTCTAGATACCG	To amplify *aac(3)IV* for *SCO1678* replacement
SCO1678_acc_r	TCCTCGACTGACGACGTCTCCCCCTGCCGCCCCGGCGCTCAAACAAAAGCTGGAGCTC	
SCO1678NcoI-f	AATTAACCATGGGCAGCACACCGGGCCGGGGGCT	*SCO1678* ORF for protein production
SCO1678HindIII-r	AATTAAAAGCTTGGGCGCCAGGATGTCCAGCT	
SCO1678gusXbaI	AATAATCTAGACAGGGGACCGATGGTGGTCT	Promoter region of *SCO1678* to clone into pGUS
SCO1678gusKpnI	AATAAGGTACCGCACTCAGCGTACGGGGCAT	
SCO1678 cmpl-f	AATAAGGATCCAGGGGACCGATGGTGGTCTT	SCO1678 with 500 bp promoter
SCO1678 cmpl-r	AATAATCTAGATTCCTCGACTGACGACGTCT	
SCO1679Cy5-f	AGCCAGTGGCGATAAGCTGTGATCGCGGGGCCGAGG	Cy5-labeled *gntR-K* intergeneric region
SCO1679Cy5-r	AGCCAGTGGCGATAAGGTTGCTGCATCGCACTCTCG	
SCO1680Cy5-f	AGCCAGTGGCGATAAGGCAGCCGGACGAGGCGGGAG	Cy5-labeled *gntP* promoter region
SCO1680Cy5-r	AGCCAGTGGCGATAAGGGTGGTTCCCTTGCGGTGAT	
SCO1681Cy5-f	AGCCAGTGGCGATAAGCTGGCTGGTGAAGGAGTACT	Cy5-labeled *gntZ* promoter region
SCO1681Cy5-r	AGCCAGTGGCGATAAGCGTCGTACTCCTGTTCCTGC	
SCO1263Cy5-f	AGCCAGTGGCGATAAGCAGGGCCAGCAGACCGCCCA	Cy5-labeled *SCO1263* promoter
SCO1263Cy5-r	AGCCAGTGGCGATAAGCGCTCCATCCCAGCGGACGC	
RTSCO1678-f	TGCGAGTGGAACGTCTACGA	RT-PCR analysis of *SCO1678*
RTSCO1678-r	GTCCTCGAACATCACGTCGT	
RTSCO1679-f	AACATCGCCAAGATGACGGC	RT-PCR analysis of *SCO1679*
RTSCO1679-r	GCCCGTTCGGTGATCTCCTC	
RTSCO1680-f	TGTTCTTCGAGGTCGGCATC	RT-PCR analysis of *SCO1680*
RTSCO1680-r	CTTGAGCAGCATCAGCACGA	
RTSCO1681-f	TCGACATCCTGGTCAACAAC	RT-PCR analysis of SCO1681
RTSCO1681-r	GGTGCTGAACTCCTCGTCCT	
RTSCO4991-f	ACCGGTCTGATCTTCGGCAT	RT-PCR analysis of *SCO4991*
RTSCO4991-r	ATCCAGGCGGAGAACACCCA	
RTgntR-K_f	ACTCGACGAGGTGCATGGAC	RT-PCR of *gntR-K* intergeneric region
RTgntR-K_r	GCCGTCATCTTGGCGATGTT	
RTgntK-P_f	TGATCGAGGACCGGATGTCG	RT-PCR of *gntK-P* intergeneric region
RTgntK-P_r	TGGTGATGACCTTGTCCAGC	
RTgntP-Z_f	ACATGTCGACCACGCACGC	RT-PCR of *gntP-Z* intergeneric region
RTgntP-Z_r	ACCGTCGGTCACGTCGAACA	
RThrdB_f	CGAGGACGAGGCGACCGAGGAG	RT-PCR analysis of *hrdB* gene
RThrdB_r	CAGCTTGTCCTCGGCGAACAGA	
